# A longitudinal cohort study of watch and wait in complete clinical responders after chemo-radiotherapy for localised rectal cancer: study protocol

**DOI:** 10.1186/s12885-022-09304-x

**Published:** 2022-03-01

**Authors:** Sina Vatandoust, David Wattchow, Luigi Sposato, Michael Z Michael, John Leung, Kirsten Gormly, Gang Chen, Erin L. Symonds, Jeanne Tie, Lito Electra Papanicolas, Susan Woods, Val Gebski, Kelly Mead, Aleksandra Kuruni, Christos S. Karapetis

**Affiliations:** 1 Flinders Medical Centre, Adelaide, Australia; 2 Flinders Centre for Innovation in Cancer, Flinders University, Flinders Drive, Bedford Park, Adelaide, South Australia 5042 Australia; 3 GenesisCare, Adelaide, Australia; 4 Dr Jones & Partners Medical Imaging, Adelaide, Australia; 5 University of Adelaide, Adelaide, Australia; 6 Monash University, Melbourne, Australia; 7 The Walter and Eliza Hall Institute of Medical Research, Melbourne, Australia; 8 Peter MacCallum Cancer Centre, Melbourne, Australia; 9 University of Melbourne, Melbourne, Australia; 10 South Australian Health and Medical Research Institute, Adelaide, Australia; 11 NHMRC Clinical Trials Centre, The University of Sydney, Sydney, Australia

**Keywords:** Rectal Neoplasms, Chemoradiotherapy, Neoadjuvant Therapy, Watchful Waiting, Treatment Outcome, Patient Reported Outcome Measures, Biomarkers, Quality of Life, Health Economics

## Abstract

**Background:**

Rectal Cancer is a common malignancy. The current treatment approach for patients with locally advanced rectal cancer involves neoadjuvant chemoradiotherapy followed by surgical resection of the rectum. The resection can lead to complications and long-term consequences.

A clinical complete response is observed in some patients after chemoradiotherapy. A number of recent studies have shown that patients can be observed safely after completing chemoradiotherapy (without surgery), provided clinical complete response has been achieved. In this approach, resection is reserved for cases of regrowth. This is called the watch and wait approach. This approach potentially avoids unnecessary surgical resection of the rectum and the resulting complications. In this study, we will prospectively investigate this approach.

**Methods:**

Adult patients with a diagnosis of rectal cancer planned to receive neoadjuvant long course chemoradiotherapy (± subsequent combination chemotherapy) will be consented into the study prior to commencing treatment. After completing the chemoradiotherapy (± subsequent combination chemotherapy), based on the clinical response, subjects will be allocated to one of the following arms: subjects who achieved a clinical complete response will be allocated to the watch and wait arm and others to the standard management arm (which includes resection). The aim of the study is to determine the rate of local failure and other safety and efficacy outcomes in the watch and wait arm. Patient reported outcome measures and the use of biomarkers as part of the clinical monitoring will be studied in both arms of the study.

**Discussion:**

This study will prospectively investigate the safety of the watch and wait approach. We will investigate predictive biomarkers (molecular biomarkers and imaging biomarkers) and patient reported outcome measures in the study population and the cost effectiveness of the watch and wait approach. This study will also help evaluate a defined monitoring schedule for patients managed with the watch and wait approach. This protocol covers the first two years of follow up, we are planning a subsequent study which covers year 3–5 follow up for the study population.

Trial registration.

Name of the registry: Australia and New Zealand Clinical Trials Registry (ANZCTR). Trial registration number: Trial ID: ACTRN12619000207112 Registered 13 February 2019,https://www.anzctr.org.au/Trial/Registration/TrialReview.aspx?id=376810

**Supplementary Information:**

The online version contains supplementary material available at 10.1186/s12885-022-09304-x.

## Background

Rectal Cancer is a common malignancy [1], comprising one third of all colorectal cancer cases [2]. In patients with non-metastatic locally advanced rectal cancer (stage II or III), the current standard management approach involves pre-operative (neoadjuvant) chemotherapy and radiotherapy followed by total mesorectal excision. Neoadjuvant chemoradiotherapy (CRT) is also recommended in patients with extramural vascular invasion (EMVI)—detected by magnetic resonance imaging (MRI)—as EMVI has been identified as a risk factor that predicts relapse, regardless of T and N stage.[3] Surgical resection of the rectum involves a mortality risk and can lead to considerable morbidity, including serious complications such as anastomotic leak.[4, 5] Other possible complications following rectal cancer resection include sexual and urinary dysfunction, which can occur in up to 25% of patients treated by radical surgery, even with meticulous nerve-sparing procedures and in highly specialized centres.[6].

Preoperative CRT can reduce the size of the primary tumour and the depth of tumour penetration and can potentially sterilize the involved lymph nodes. Large randomized studies have established preoperative CRT as the preferred treatment option for patients with stage II or III rectal cancer.[7–11] More recently, studies have shown that the addition of combination chemotherapy to either a short course of radiotherapy or standard long course concurrent CRT before surgery, can improve outcomes.[12–14] This approach is called total neoadjuvant therapy (TNT).

In some patients, preoperative CRT can lead to pathological complete response. Pathological complete response is established when no viable malignant cells are found in the resected surgical specimen. Patients who achieve pathological complete response have better outcomes.[15] Pooled analysis of data shows the rate of pathological complete response to be approximately 15% with CRT and 30% with TNT.[16] Establishing pathological complete response requires examination of the surgical specimen. In patients who have not had surgery, clinical complete response (cCR) is used as a surrogate for pathological complete response. cCR is established when no malignancy is found on clinical examination, imaging, endoscopy (sigmoidoscopy) and biopsy.[15, 17] Surgery may not be necessary in these patients. This approach is known as watch and wait.

Accumulating data suggest that watch and wait in patients with cCR might be a safe option. Habr-Gama and colleagues were the first to report on series of patients treated in line with the watch and wait approach.[18] They included patients with mid to distal locally advanced rectal cancers. Patients were assessed for clinical response 8–10 weeks after completion of CRT, and those with residual tumour were advised to have surgery. Those with cCR were monitored closely for an additional 10 months. Patients who had a sustained cCR at one-year post CRT were offered non-operative management. Ninety patients were managed with this watch and wait approach. After a median follow up of 60 months they reported 94% rate of local disease control and 78% organ (rectal) preservation. From the 90 patients, 28 (31%) developed local recurrence. Of the 28 patients with local recurrence, 26 (93%) were managed with salvage surgery and 6 (7%) had unsalvageable local recurrence (local failure). All cases of local failure happened in the first two years of follow up.[18] Since then, other groups have also published results of retrospective studies of patients managed with this approach.[19–23] Until recently the evidence supporting the watch and wait approach was based on small retrospective studies. The international watch & wait database (IWWD) has recently been established to study this approach in a large registry of pooled individual patient data.[24] The application of combination chemotherapy, either before or after a ‘standard’ course of concurrent CRT, has been studied in a randomised phase II trial that examined this strategy as part of a watch and wait approach for those that achieved cCR. In this study, patients with stage II and III rectal cancers were assigned to either an induction group, where patient received 4 months of chemotherapy (FOLFOX or CAPOX) followed by CRT or to the consolidation group where patients received CRT followed by 4 months of chemotherapy. Patients with incomplete clinical response proceeded to surgery and patients with cCR were assigned to a watch and wait protocol. Promising preliminary results have been presented from this study, supporting the watch and wait approach.[14] Final results of this study are awaited.

We designed the current study to prospectively investigate the safety of the watch and wait approach and to study multiple secondary outcomes which have not been studied adequately in the past. These secondary outcomes include biomarker studies, patient reported outcome measures (PROMs) and cost effectiveness.

Further research is necessary to discover and validate diagnostic biomarkers of cCR and predictive biomarkers of local failure. In this study we are aiming to investigate a multitude of biomarkers, including imaging biomarkers, non-coding RNAs [25, 26], circulating tumour DNA (ctDNA) [27–29], circulating methylated DNA [30, 31], tumour infiltrating lymphocytes (TILs) [32–34], organoids [35] and gut microbiome [36–38]. PROMs have not been studied in detail in this group of patients. In this study, we will thoroughly explore the burden of long-term side effects as well as other quality of life measures using validated questionnaires. We will use specific tools to investigate bowel related quality of life measures, including incontinence and bowel function. We will also study fear of cancer recurrence in the study population. Like other cancer survivors, the current study population experience ongoing issues during the survivorship continuum, we are aiming to assess these issues and the supportive care needs of the study population. Cost effectiveness evidence has not been thoroughly measured and reported for the watch and wait approach in patients with rectal cancer. The scarcity of resources mandates that health care investments be made in the most cost-effective manner. Health economics evaluation is a method whereby benefits and costs of alternative treatment options can be considered to aid decision-makers in prioritising and allocating health resources.[39].

## Methods

### Aims and objectives

#### Primary objectives

1. To determine the safety and efficacy of the watch and wait approach, by measuring the following endpoints in the watch and wait arm:
  - Co-Primary endpoint 1: The ‘two-year local failure rate’
  - Co-Primary endpoint 2: The rate of rectal preservation

Secondary objectives:

1. To determine the safety of the watch and wait strategy by measuring the following endpoints (in the watch and wait arm):
  - The ‘two-year local regrowth rate’
  - The ‘two-year distant metastasis rate’
  - The overall survival
  - The ‘incurable disease-free survival rate’ (recurrent cancer that cannot be surgically excised with the intention of achieving a cure)
2. Evaluate the role of biomarkers
  - Plasma and tumour biopsy biomarkers (non-coding RNAs, methylated DNA), TILs, organoids and gut microbiome:
    i) To predict cCR (in both arms)
    ii) To identify residual disease, micrometastases and recurrence risk (in both arms)
    iii) As prognostic biomarkers (in both arms)
  - Imaging biomarkers (mrTRG, diffusion and T2 signs)
    i) To predict sustained cCR
3. PROMs (in both arms)
  - Quality of life (quality of life and health related quality of life)
  - Bowel related quality of life
  - Fear of cancer recurrence
  - Supportive care needs
4. To evaluate a defined monitoring schedule in the watch and wait arm
5. Cost effectiveness analysis: (both arms)

### Design

This is a Longitudinal Cohort Study.

Eligible subjects, after consent/registration, will complete CRT and then based on response will be allocated to one of the two study arms.

### Subect population

#### Target population

Adult patients with a diagnosis of locally advanced rectal cancer who are going to receive combined long course CRT (± subsequent combination chemotherapy).

#### Study setting

Participants will be accrued from hospitals and outpatient clinics in Australia. List of s study sites can be found through: https://www.anzctr.org.au/

Inclusion criteria.

1. Age ≥ 18 years
2. Biopsy proven locally advanced rectal adenocarcinoma:
  i) Locally advanced disease defined as: T3 N0-2, T1-2 N1-2 [Based on AJCC UICC 2017]
3. Subject to undergo long course neoadjuvant CRT (± subsequent combination chemotherapy) based on a multidisciplinary meeting recommendation
4. Considered suitable for long course pelvic radiation therapy
5. Considered suitable for surgery
6. Considered suitable for MRI
7. Willing and able to comply with all study requirements, including treatment and follow up assessments
8. Signed, written informed consent

Exclusion criteria.

1. Presence of metastatic disease (M1)
2. T4 disease based on AJCC 2017
3. Local recurrence of previously treated rectal cancer
4. Previous pelvic radiotherapy
5. Contraindication to fluoropyrimidine chemotherapy
6. History of another malignancy:
  i) Patients with a history of adequately treated carcinoma-in-situ, basal cell carcinoma of the skin, squamous cell carcinoma of the skin, or superficial transitional cell carcinoma of the bladder are eligible. Patients with a history of other malignancies are eligible if they have been continuously disease free for at least 5 years after definitive primary treatment.
7. Concurrent illness, including severe infection that may jeopardize the ability of the patient to undergo CRT with reasonable safety
8. Presence of any psychological, familial, sociological or geographical condition potentially hampering compliance with the study protocol including CRT and/or follow-up schedule
9. Pregnancy or lactation

### Study procedures

#### Screening

Written informed consent (model consent form: supplementary file-1) will be obtained before screening procedures are undertaken. Participants will have pretreatment sigmoidoscopy. Pre-treatment specimens (including biopsy, plasma and stool specimens) will be collected within this time-frame. If all screening procedures are performed and eligibility is confirmed, the patient will be registered for the trial.

#### Registration

Subjects must meet all the inclusion criteria and none of the exclusion criteria to be eligible for this study. Subjects must be registered before entering the study. Registration should be done only after all screening assessments have been performed.

Run-in phase

- Participants receiving only CRT:
  i) Run-in phase post CRT – week 8–10: patients will have a clinical assessment and endoscopy (sigmoidoscopy) and CT scan (run-in investigations) between week 8—10 post CRT. If there is no evidence of residual cancer found in the run-in phase investigations, subjects will have an MRI.
  ii) Allocation visit – week 8–10: At the allocation visit all investigations during the run-in phase will be evaluated. Patients who have achieved cCR will be allocated into the watch and wait arm. All other patients will be allocated to standard management arm.
- Participants receiving TNT:
  i) Run-in phase post TNT – week 2–3: patients will have a clinical assessment and endoscopy and CT scan (run-in investigations) between week 2–3 after the last dose of TNT. If there is no evidence of residual cancer found in the run-in phase investigations, subjects will have an MRI. If patients are required or decide to stop the doublet chemotherapy earlier than the recommended number of cycles, they will proceed to tumour response assessment 2–3 weeks after the last dose of doublet chemotherapy and allowing a minimum of 8 weeks post CRT before the response evaluation takes place.
  ii) Allocation visit (participants receiving TNT) – week 2–3 after the last dose of doublet chemotherapy. At the allocation visit all investigations during the run-in phase will be evaluated. Patients who have achieved cCR will be allocated into the watch and wait arm. All other patients will be allocated to standard management arm.

### Treatment Plan

#### Administration of study treatments

Radiotherapy

- Radiation therapy is to start with concurrent chemotherapy.
- Simulation with bladder and bowel protocol.
- Immobilisation with ankle and knee supports.
- Anal marker is optional.
- Fusion of MRI preferred with planning CT scan at 2–3 mm slices through relevant area.
- Volumetric modulated arc therapy (VMAT)/Intensity modulated radiotherapy (IMRT) is the preferred technique with a simultaneous integrated boost.
- Target volumes:
  Primary –
- Gross tumour volume of primary (GTV-P) = gross disease
- Clinical target volume of primary (CTV-P) = GTV-P plus adequate margin superior and inferior (2cm), radially (1cm but discretion of clinician).
- Planning target volume of primary (PTV-P) = CTV-P + 0.5 -1.0 cm.
  Nodes
- Gross tumour volume of lymph nodes (GTV-N) = gross nodal disease.
- Clinical target volume of lymph nodes (CTV-N) = GTV-N plus mesorectum, internal iliac, presacral and other appropriate nodal areas as designated by radiation oncologist e.g., if anal canal involvement, the external iliac, obturator, inguinal and ischiorectal fossa nodes may be treated.
- Planning target volume of lymph nodes (PTV-N) = CTV-N plus 0.5- 0.7 cm.
- Dose prescription: PTV-P and PTV-N = 45 Gy /25F / 1.8 Gy per fraction
- Simultaneous integrated boost to gross disease for total of 50 Gy /25F.
- A one phase technique to 50.4 Gy /28F or 50 Gy /25F is optional.
- Image verification: cone-beam computed tomography (CBCT) day1 to day 3 then weekly as a minimum.

### Chemotherapy

Neoadjuvant chemotherapy will be given concurrently with radiation and will comprise of a single agent fluoropyrimidine regimen: intravenous 5-FU: 225 mg/m2 continuous IV infusion via pump during the radiation therapy course, OR Oral capecitabine—825 mg/ m2 PO BD (days 1–5, excluding weekend).

Proceeding with ‘adjuvant’ chemotherapy (i.e., chemotherapy after completion of concurrent CRT) in the watch and wait arm is based on treating clinicians’ decision. In the watch and wait arm, it is recommended that patients receive adjuvant doublet chemotherapy with a fluoropyrimidine and oxaliplatin (for a total of 3 months (6 cycles of FOLFOX or 4 cycles of CAPOX) if the patient did not receive TNT and they are otherwise considered suitable for chemotherapy.

For patients considered suitable for TNT, combination doublet chemotherapy will commence 10–14 days after completion of CRT. This will consist of either 8 cycles of FOLFOX, administered on 2-week cycles, or 6 cycles of CAPOX, administered on 3-week cycles. Doublet chemotherapy dosed as per local and eviQ guidelines (eVIQ guidelines are available at https://www.eviq.org.au). If patients are required or decide to stop the doublet chemotherapy earlier than the recommended number of cycles, they will immediately proceed to tumour response assessment, allowing a minimum of 8 weeks post CRT before the response evaluation takes place.

Chemotherapy dose modifications are in accordance with local and eviQ guidelines.

Concomitant medications/treatments during chemotherapy:

- Metronidazole and warfarin are best avoided during CRT with 5-FU and must be used with caution. Warfarin is best avoided during CRT with capecitabine, and must be used with caution
- Folic Acid should not be used during CRT with 5-FU.
- If necessary, antibiotics can be used during CRT, but their use must be documented and captured in the database for evaluation.

### Assessment plan

Schedule of assessments is summarized in Table 1. Schedule of PROMs is summarized in Table 2.

- Timing of the monitoring visits will be calculated from the allocation visit.
- The watch and wait arm: monitoring visits: monitoring visits and procedures to be scheduled according to the assessment timetable (± 2 weeks)
- The standard management arm: PROMs questionnaires to be completed according to the to the assessment timetable (± 2 weeks)
- Follow-up after stopping the study: If a patient wishes to stop the study visits, they will be requested to allow their ongoing health status to be periodically reviewed via continued study visits or phone contact or from their general practitioner, or medical records, state-based cancer registries and/or the national mortality registry.
- For patients who have been lost to follow-up, Medicare may be used to provide updated contact information and/or hospitalisations and the national registry may be used to collect mortality information.

### Outcomes, endpoints and other measures

#### Safety and efficacy

Two-year local failure rate (Co-Primary Endpoint)

- This endpoint will be measured in the watch and wait arm only. It is defined as local recurrence (i.e., recurrence in the region of the rectum, mesorectum and adjacent lymph nodes) that cannot be resected with clear margins.
- Metastatic disease:
- Metastatic disease in the presence of local recurrence is considered ‘local failure’
- Metastatic disease in the absence of local cancer recurrence is not considered ‘local failure’
- The rate of rectal preservation (Co-Primary endpoint)
- This endpoint will be measured in the watch and wait arm only. It is defined as the rate of organ (rectal) preservation at two years.

Rate of incurable-disease free survival (incurable-disease or death)

- Defined as the interval from date of registration to the date of first evidence of ‘local failure’ or ‘distant metastatic disease’ or death, whichever occurs first
  Overall survival (death from any cause)
- Overall survival is defined as the interval from the date of registration to date of death from any cause, or the date of last known follow-up alive.
  Two-year local recurrence rate
- Local Recurrence is defined as evidence of recurrent disease within the pelvis, found by examination, endoscopy or imaging (CT or MRI). Biopsy and pathological confirmation are encouraged if technically possible and safe.
  Two-year distant recurrence rate
- Distant Recurrence is defined as evidence of recurrent disease other than local recurrence, found by imaging or on examination.

Translational research (biomarkers).

### Non-coding RNA

Pre-treatment biopsies will be collected from all patients. Total RNA will be extracted, and the quality evaluated. A two-stage approach will be adopted to identify micro-RNAs that are associated with response of rectal tumours to CRT. In the discovery stage Next-Generation Sequencing (NGS) will be used to compare micro-RNA expression within 20 patients in each arm. In the validation stage, we will employ RT-PCR assays to determine the relative micro-RNA levels in a larger set of tissue specimens with known clinical outcomes (N = 35 in each arm). Correlations will be established between the levels of a defined micro-RNA panel and therapeutic response.

### ctDNA

The study will assess the impact of ctDNA in predicting recurrence in patients in the watch and wait arm: original tumour biopsy tissue will be analyzed with whole exome sequencing for somatic variants. The identified variants will be queried and quantified in plasma using the SaferSeqS assay.[41] We plan to explore baseline ctDNA levels as prognostic markers and change in levels over time as lead indicators of cancer recurrence.

### Methylated DNA

Blood will be collected prior to neoadjuvant treatment and at defined time-points from patients in the watch and wait arm. Methylated *BCAT1* and *IKZF1* will be studied as previously described.[42–44] The prognostic influence of ctDNA methylation will be determined through correlation of methylation and clinical outcomes (response, recurrence and survival). Marker evaluation pre and post CRT will also be assessed and correlated with cancer recurrence in the watch and wait arm. Emergence of methylated DNA after previous clearance will also be correlated with clinical cancer recurrence, and this marker may provide an early indication of cancer persistence or regrowth, ahead of imaging changes.

### TILs

The role of TILs in predicting response to treatment will be assessed. Pre-treatment specimens will be collected. TILs will be measured based on the methodology proposed by the International Immunooncology Biomarkers Working Group. [32, 33] The correlation between TILs and response to neoadjuvant treatment will be assessed.

### Microbiome

The gut microbiome will be evaluated as a predictor of response to CRT. Stool specimens will be collected from participants before starting CRT and after finishing CRT. We will use 16S rRNA gene amplicon sequencing to assess the microbiome as previously described.[45] We will then investigate the correlation between microbiome characteristics (including composition and diversity) and clinical outcomes (including response to neoadjuvant therapy).

### Organoids

A biopsy will be collected from pre-treatment colonoscopy. Organoids will be grown from the biopsy as previously described.[46] These organoids will be expanded for 2 weeks and then subjected to 5-FU as the common agent used in CRT. This will be followed by gamma-irradiation to complete the CRT protocol, mimicking standard neoadjuvant CRT for rectal cancer. Cell viability will be measured generating an ‘area under the curve’ (AUC) value for each organoid in response to CRT. We will then determine whether organoid culture can be used as a biomarker to predict sensitivity to neoadjuvant treatment for rectal cancer patients. We will also use the established cultures for future basic and translational research.

### PROMs

#### Quality of life

Quality of life will be measured using the EORTC QLQ-C30 (ver-3.0) and EORTC QLQ-CR-29 (ver-2.1) questionnaires. EORTC QLQ-C30 (ver-3.0) is a validated questionnaire developed to assess the quality of life of cancer patients.[47] QLQ-CR-29 is a validated questionnaire developed to assess the quality of life of patients with a history of colorectal cancer and is used in addition to the EORTC QLQ-C30 questionnaire.[48].

#### Health related quality of life

Health-related quality of life is a multi-dimensional concept that includes domains related to physical, mental, emotional and social functioning. This outcome will be measured using a validated questionnaire: the EQ-5D-5L questionnaire. This is a widely used generic preference-based tool to measure the health-related quality of life and is commonly used to elicit health utility scores for health economic evaluation.[49].

#### Bowel related quality of life

This outcome encompasses multiple aspects of bowel function and will be measured using the Memorial Sloan Kettering cancer center bowel function instrument (MSKCC BFI)[50] and low anterior resection syndrome score (LARS score)[51].

#### Fear of cancer recurrence (FCR)

FCR is defined as feeling anxious about cancer coming back and is measured by a shorter form of the fear of cancer recurrence inventory- short form (FCRI-SF). FCRI-SF is a tool used for e screening of clinical levels of FCR.[52] To interpret the FCR results, and to measure response efficacy we will use a modified response efficacy scale specifically designed for this study. (Response efficacy scale: supplementary file-3).

#### Survivorship and supportive care needs

Ongoing supportive care needs across the survivorship continuum will be measured using cancer survivors' unmet needs measure (CaSUN). This is a tool, developed to evaluate a self-reported measure of cancer survivors’ supportive care needs.[53].

#### Cost effectiveness

To determine the cost effectiveness of the watch and wait approach, a within-trial economic evaluation from a healthcare sector perspective will be conducted using a cost-utility analysis (CUA), in which the effectiveness will be measured by quality-adjusted life-years (QALYs). The cost-utility analysis involves estimating the incremental cost effectiveness ratios (ICERs) of the watch and wait approach versus standard management plan. For the within-trial economic evaluation, only costs and effects that accumulate within the trial participation up to 24 months will be considered.

#### Cost-utility analysis

In the cost-utility analysis, the effectiveness will be measured by using the QALYs (which considers both the quantity and quality of life). The health state utilities (which indicates the ‘Q’ in QALYs) will be firstly estimated based on the EQ-5D-5L questionnaire and scored using Australian-specific tariff. Alternatively, the health state utility generate from a cancer-specific quality of life instrument QLQ-C30 will also be considered to investigate the robustness of the results using a recently published Australian-specific tariff.[54] Resource use and total costs associated with the two management arms will be populated based on the data collected from the trial. Key resource use items will include rectal cancer treatment costs (such as surgery, imaging, chemotherapy, and endoscopy), and any associated healthcare costs. Both costs and benefits will be discounted by 5% in the base model, in line with government policy. Decision uncertainty will be summarised through the presentation of cost effectiveness acceptability curves.

### Safety reporting

#### Definitions

Adverse event (AE) is any untoward medical occurrence in a patient or clinical investigational subject, and which does not necessarily have a causal relationship with the management approach (MRI, endoscopy and surgery).

For this trial, only adverse events deemed related to the study procedures (MRI, endoscopy and surgery) will be collected and reported on.

A serious adverse event (SAE) is any untoward medical occurrence that:

- results in death,
- is life-threatening (i.e., the subject is at risk of death at the time of the event),
- requires inpatient hospitalisation or prolongation of existing hospitalisation,
- results in persistent or significant disability or incapacity,
- is a congenital anomaly/birth defect
- other important medical events which, in the opinion of the investigator, are likely to become serious if untreated, or as defined in the protocol.

#### Reporting of SAEs

The investigator is responsible for reporting all SAEs occurring during the study to the principal investigators within 1 working day of the investigator becoming aware of the event using the SAE form. SAEs must be reported up to 30 days from the end of study intervention. The principal investigators must notify the local Human Research Ethics Committees (HREC) as required.

#### Central review and specimen collection

Central tissue collection: paraffin-embedded tissue blocks will be collected for central histology review and for translational studies. Fresh tissue will be collected for biomarker studies.

Central blood collection: blood collection is required for all study patients. Serum and plasma for biomarkers will be collected and initially processed at each site. The frozen samples will be sent to a central lab for analysis.

Central imaging collection: Central Review: MRI scans will be reviewed centrally.

Other central collection: stool specimens will be collected and will be studied centrally to assess for gut microbiome. If there are available samples which have been collected and stored and they meet the sample requirements per the lab manual, these may be used in addition to or in place of collecting new samples.

### Statistical considerations

#### Sample size

The sample size is determined by utilizing the precision of the expected rate of local failure for patients with cCR after CRT. Based on available literature, the failure rate by 3-years is assumed to be 5%. A sample of 50 participants with cCR will be required to allow for 47 assessable participants (considering 5% potential drop out). This sample size calculation provides a precision of ± 10% with 95% confidence for the estimate of 5% local failure rate. Expecting 20% rate of cCR, this will translate into a total of 250 participants to be enrolled into the study to result in 50 participants in the watch and wait arm. (50 participants in the watch and wait arm and 200 in the standard management arm).

### Statistical analysis

Analyses will include all patients enrolled. Local control will be assessed by the proportion, together with the 95% confidence interval, of patients not failing locally as their first event at 2 years. This proportion will be estimated using a competing risk cumulative incidence curve where distant failure or death prior to any failure is considered as a competing risk. This will be estimated in both groups.

Time-to event endpoints of progression free, local control and overall survival will be described using the method of Kaplan–Meier or cumulative incidence. Formal comparisons between groups will be performed using logrank, Cox proportional hazards, Gray and Fine & Gray methods. Exploratory comparative analyses include univariate and multivariate regression models for time-to-event outcomes, regression models for continuous outcomes and logistic regression analyses for binary outcomes. Other exploratory analyses will also be performed as appropriate. No imputation for missing data is envisaged which will be assumed to be missing at random. Quality of life will be summarized using trajectories over the follow-up time.

In the watch and wait arm: the study will be stopped if the number of cases with local failure reaches 3 cases in the first 15 participants, and 5 cases in the first 30 participants at 3 years based on the charts of Mehta and Cain. [55] (Table 3).

Study Committees.

Trial management committee

The trial management committee will oversee study planning, monitoring, progress, review of information from related research, and implementation of recommendations from other study committees and external bodies (e.g., ethics committees).

Independent safety and data monitoring committee

This study has an independent safety and data monitoring committee including experts in the field who are not involved in this study.

Ethics and administrative issues:

- Ethics and regulatory compliance
  This study will be conducted according to the Note for Guidance on Good Clinical Practice (CPMP/ICH/135/95) annotated with TGA comments (Therapeutic Goods Administration DSEB July 2000) and in compliance with applicable laws and regulations. The study will be performed in accordance with the NHMRC Statement on Ethical Conduct in Research Involving Humans (© Commonwealth of Australia 2007), and the NHMRC Australian Code for the Responsible Conduct of Research (©Australian Government 2007), and the principles laid down by the World Medical Assembly in the Declaration of Helsinki 2008.
  To this end, no patient will be recruited to the study until all the necessary approvals have been obtained and the patient has provided written informed consent. Further, the investigator shall comply with the protocol, except when a protocol deviation is required to eliminate immediate hazard to a subject. In this circumstance the principal investigator and HREC must be advised immediately.
- Protocol amendments
  Changes and amendments to the protocol can only be made by the trial management committee. Approval of amendments by the institutional HREC is required prior to their implementation. In some instances, an amendment may require a change to a consent form. The Investigator must receive approval/advice of the revised consent form prior to implementation of the change. In addition, changes to the data collected, if required, will be incorporated in the amendment.
- Confidentiality

The study will be conducted in accordance with applicable privacy acts and regulations. All data generated in this study will remain confidential. All information will be stored securely at the clinical trials unit and will only be available to people directly involved with the study and who have signed a confidentiality agreement.

- Data handling and record keeping
  All trial data required for the monitoring and analysis of the study will be recorded on the case report forms (CRF). All required data entry fields must be completed. Data corrections will be done according to the instructions provided. The investigator will be asked to confirm the accuracy of completed CRFs by signing key CRFs as indicated. All study-related documentation will be maintained for 15 years following completion of the study.
- Study Monitoring
  Data from this study will be monitored by clinical trials unit or their delegates. Monitoring will include centralised review of CRFs and other study documents for protocol compliance, data accuracy and completeness.
- Publication policy

The trial management committee will appoint a writing committee to draft manuscript(s) based on the trial data. Manuscript(s) will be submitted to peer-reviewed journal(s). All publications must receive prior written approval from the principal investigators prior to submission.

## Discussion

In this study we are aiming at investigating the safety of the watch and wait approach in patients who achieve cCR after CRT for locally advanced rectal adenocarcinoma. The current study will add to accumulating data from previous retrospective series and ongoing prospective studies. We will investigate a multitude of biomarkers to assess their role in predicting response or treatment failure. This will help identify the group of patients who benefit from this approach. This study investigates a comprehensive set of PROMs and helps understand the watch and wait approach from the patients’ perspective. We will investigate the cost effectiveness of the treatment; the findings will help guide health policy. Finally, this study will help establish a structured follow up protocol for patients managed by the watch and wait approach in the future.

Retrospective studies of the watch and wait approach are prone to selection bias. The ideal study design to avoid this is a randomized study comparing the watch and wait approach with standard treatment (surgery) in patients who achieve complete response; however, randomizing patient to a surgical approach versus non-surgical management is challenging and is unlikely to accrue the required number of patients. To avoid selection bias, we have elected to enrol the participants into the study prior to starting CRT: the watch and wait approach is offered to the participant who achieve cCR. This way we are aiming to reduce the selection bias.

To avoid heterogeneity of the study population, and improve the reliability of the results, we have included a well-defined population of patients with specific disease stage. Patients with cT2 N0 without EMVI involvement on MRI, are excluded from the study. In this group of patients, long course CRT is not considered the standard of care in many centres. Although some of these patients may receive long course CRT and achieve a complete response, routine neoadjuvant CRT involves the risk of “over-treatment” in many of these patients. Patients with T4 tumours are also excluded from the study. By definition, T4 tumours invade the visceral peritoneum or adjacent organs or structures. During the study design investigators chose to exclude this sub-group, as they potentially have a higher risk of recurrence and with the watch and wait approach they may be at risk of under-treatment. Another sub-group of patients who are not included in this study are patients with metastatic disease. Some of the important outcomes of the current study are the rate of treatment failure, disease free survival and overall survival. Presence of metastatic disease affects these crucial outcomes, and therefore patients with metastatic disease are excluded from the study.

The current study design includes two years of follow up. Although retrospective data has shown that the majority of local regrowth and recurrences happen during the first two years [24], local regrowth or recurrence can happen later and longer follow up is essential. We are currently designing a study focusing on further follow up of up to five years for the current study population.

**Table 1.** Schedule of assessments

All Participants		Month	Consent	Clinical Assessment	Sigmoidoscopy	Biopsy	CT scan	MRI	Stool Test	Blood test	Outcome events	PROMs
	Screening visit		X	X	X^*^	X	X		X	X		X
	Chemoradiotherapy											
	Run-in phase^**^			X	X	X	X	X^^^	X	X		X
	Allocation visit^**^	0										
Watch and Wait Arm	Visit	Month										
	1	3		X	X	When clinically indicated		X		X	X	
	2	6		X	X		X	X		X	X	
	3	9		X	X			X		X	X	
	4	12		X	X		X	X		X	X	X
	5	15		X	X						X	
	6	18		X	X			X		X	X	
	7	21		X	X						X	
	8	24		X	X		X	X		X	X	X
Standard Management Arm	Visit	Month										
	1	3			As per standard of care						X	
	2	6									X	
	3	9									X	
	4	12									X	X
	5	18									X	
	6	24									X	X

^a^ Screening includes either a sigmoidoscopy or a colonoscopy as recommended by the treating surgeon

^b^Allocation phase includes Run-in phase (6–8 weeks after finishing CRT) and allocation visit (8–10 weeks after finishing CRT)

^2nd MRI only if CT scan and endoscopic evaluation indicate Ccr

**Table 2.** Schedule of assessments: Patient Reported Outcome Measures

All Participants	Month	SCQ	EORTC QLQ-C30	CR29 EORTC QLQ	EQ-5D-5L	MSKCC BFI	LARS	FCRI-SF	Questions Response efficacy	CaSUN
Screening visit		X	X	X	X	X	X	X		
Chemoradiotherapy										
Run-in phase		X	X	X	X	X	X	X		
Allocation visit	0									
12-month visit	12	X	X	X	X	X*	X*	X	X	X
24-months visit	24	X	X	X	X	X*	X*	X	X	X

^a^Only if participant does not have a stoma

**Table 3.** .

% Local failure rate	Sample size	Stop the study if the number of local failure cases is equal to or more than:
15%	15	3
15%	30	5

## Supplementary Information

**Additional file 1.**

**Additional file 2.**

**Additional file 3.**

## Data Availability

not applicable (this is a study protocol).

## Abbreviations

5-FU: 5-Fluorouracil
AE: Adverse event
AGITG: The Australasian Gastro-Intestinal Trials Group
ANZCTR: Australia and New Zealand clinical trials registry
AUC: Area under the curve
CaSUN: Cancer survivors' unmet needs
CBCT: Cone-beam computed tomography
cCR: Clinical complete response
CRF: Case report forms
CRM: Circumferential resection margin
CRT: Chemoradiotherapy
ctDNA: Circulating tumour DNA
CTV-N: Clinical target volume of lymph nodes
CTV-P: Clinical target volume of primary
CUA: Cost-utility analysis
DWI: Diffusion-weighted imaging
EMVI: Extramural vascular invasion
FCRI-SF: Fear of cancer recurrence inventory- short form
FOV: Field of view
GTV-N: Gross tumour volume of lymph nodes
GTV-P: Gross tumour volume of primary
HR: High-resolution
HREC: Human research ethics committee
ICER: Incremental cost effectiveness ratio
IMRT: Intensity modulated radiotherapy
IWWD: The international watch & wait database
LARS: Low anterior resection syndrome
MRI: Magnetic Resonance Imaging
mrTRG: MRI tumour regression grade
MSKCC-BFI: Memorial Sloan Kettering cancer center bowel function instrument
NGS: Next-generation sequencing
PROMs: Patient reported outcome measures
PTV-N: Planning target volume of lymph nodes
PTV-P: Planning target volume of primary
QALYs: Quality-adjusted life-years
SAE: Serious adverse event
SALHN: Southern Adelaide Local Health Network Ltd
TILs: Tumour infiltrating lymphocytes
TNT: Total neoadjuvant therapy
VMAT: Volumetric modulated arc therapy
