# Supplementary material for: A longitudinal cohort study of watch and wait in complete clinical responders after chemo-radiotherapy for localised rectal cancer: study protocol
Source: BMC Cancer. 2022 Mar 1;22:222. doi: 10.1186/s12885-022-09304-x (PMC8887187; doi:10.1186/s12885-022-09304-x)
Supplement: Supplementary file 1 — Additional file 1. [file 12885_2022_9304_MOESM1_ESM.doc]

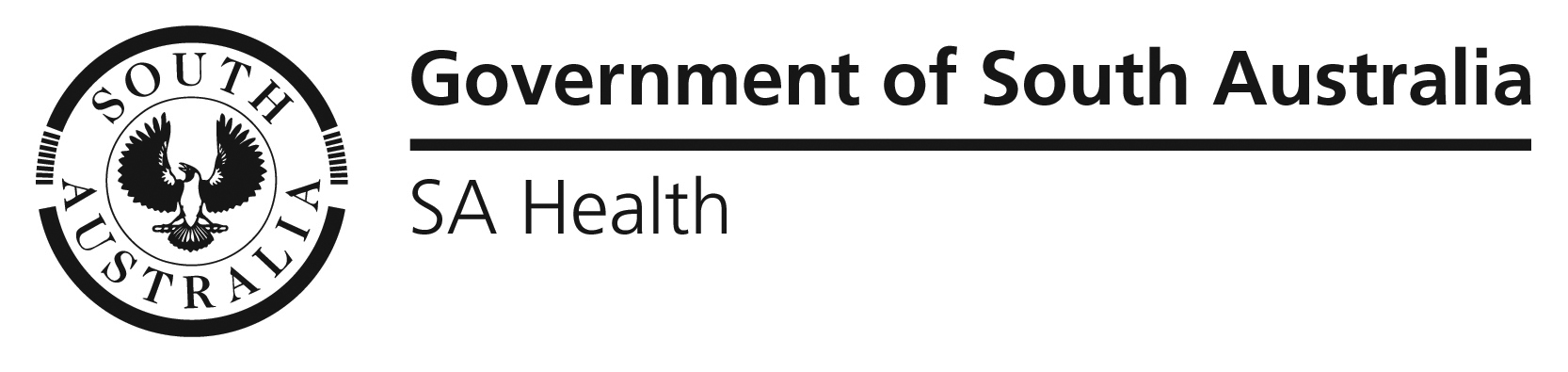


| **Participant Information Sheet/Consent Form** |
| --- |

| **Title** | A Longitudinal Cohort Study of ‘Watch and Wait’ in Complete Clinical Responders after Chemoradiotherapy for Localised Rectal Cancer |
| --- | --- |
| **Short Title** | REctal cancer Non-Operative (RENO) |
| **Protocol Number** | 2020-3 |
| **Project Sponsor** | Southern Adelaide Local Health Network Ltd (SALHN) Flinders Drive  Bedford Park SA 5042 |
| **Principal Investigator** |  |
|  |  |
| **Location** | [study site] |

**Part 1: What does participation involve?**

**1 Introduction**

You are invited to take part in this research project because you have been diagnosed with rectal cancer and your doctors recommended that you receive chemotherapy and radiation (this is called “*chemoradiation*”) followed by surgery to remove your rectum or part of your rectum.

The standard treatment (and the current best practice) includes chemoradiation followed by surgery (resection of the rectum or part of the rectum). In some patients with localised rectal cancer (approximately 2 in 5) following chemoradiation, the cancer disappears (this is called a “*complete response*”). The standard treatment includes surgery (resection of the rectum or part of the rectum); even in patients with complete response after chemoradiation. Recently, it has been shown that giving more chemotherapy for up to 4 months after the combined chemoradiation course can lead to a greater chance of achieving a complete response. The chance of getting a complete response is in the order of 40-60%. In view of this, this additional chemotherapy is now considered a standard treatment option for someone with localised rectal cancer. At the end of all the chemotherapy, the currently recommended course of action is to proceed to surgery, removing a portion of the lower bowel.

This research project is aiming to investigate an alternative approach to standard treatment for rectal cancer patients who achieve a complete response following chemoradiation with or without additional chemotherapy.

This Participant Information Sheet/Consent Form tells you about the research project. It explains the research involved. Knowing what is involved will help you decide if you want to take part in the research. Please read this information carefully. Ask questions about anything that you don’t understand or want to know more about. Before deciding whether or not to take part, you might want to talk about it with a relative, friend or local doctor.

Participation in this research is voluntary. If you do not wish to take part, you do not have to. You will receive the best possible care whether or not you take part: this includes the standard management for your disease. Depending on the stage of the disease, standard treatment for rectal cancer may include Surgery, Radiotherapy and chemotherapy and then follow-up by your treating doctors. The follow up involves clinic visits which include physical examination, and may also include blood tests, CT scans and colonoscopies.

If you decide you want to take part in the research project, you will be asked to sign the consent section. By signing it you are telling us that you:

- Understand what you have read
- Consent to taking part in the research project
- Consent to the research that is described
- Consent to the use of your personal and health information as described.

You will be given a copy of this Participant Information and Consent Form to keep.

**2 What is the purpose of this research?**

Rectal Cancer is a common malignancy. Current approach to patients with rectal cancer involves chemoradiation followed by surgery. Surgery includes the removal of rectum or part of the rectum. In about 2 in 5 patients who receive chemoradiation followed by more chemotherapy, the cancer disappears (this is called a “*complete response*”). A number of recent studies have shown that these patients can be observed safely after completing chemoradiation and the surgery (resection of rectum) can be done only if the disease reappears.

In this study, we are aiming to investigate the safety and efficacy of this approach. We are also aiming to gather information on new ways to help predict which patients will develop complete response after chemoradiation and recognise cancer earlier than it is possible with the currently available methods. We will also gather information on symptoms and other aspects of well-being, functioning, health status and quality of life (otherwise known as Patient Reported Outcome Measures) from participants.

This research has been initiated by the study doctors: Professor Christos S Karapetis, Professor David Wattchow, Dr Sina Vatandoust and Dr Luigi Sposato. Other doctors and researchers across Australia are also involved in this study.

This study is being conducted at multiple sites across Australia.

**3 What does participation in this research involve?**

The study involves the screening process, followed by chemoradiation, then further chemotherapy, then based on the disease response, participants will be allocated to one of the two study arms.

Screening visit: If you agree to participate in this study you will be asked to sign a consent form. Then you will be reviewed by the study doctors/team and assessed. This is called “*screening*”. The reason behind this process is to make sure that this study is a suitable option for you and also to ensure that you are eligible to participate in the study.

The screening process involves:

- History taking and examination: You will be asked questions about your health, and your previous medical history. Your medical records will be accessed. You will be examined by one of the study doctors (which could be your surgeon), this includes a rectal examination.
- Colonoscopy or sigmoidoscopy: The study doctor will also examine the rectum using a colonoscope. Colonoscopy is performed by inserting a device called a “*colonoscope*” into the anus to examine the lower part of the gastrointestinal tract (colon) including the lower part of the colon (rectum) (this is where the cancer is located). If you have had a recent colonoscopy, your surgeon may examine the lower part of the colon and the rectum through sigmoidoscopy. Sigmoidoscopy is performed by inserting a device called a “*sigmoidoscope*” into the anus to examine the lower part of the colon and the rectum (in contrast with colonoscopy, sigmoidoscopy does not examine the other parts of the colon). During the colonoscopy or sigmoidoscopy, pieces of tissue will be removed from your tumour (this is called a “*biopsy”*). This is in addition to the colonoscopy (or sigmoidoscopy) and biopsies that you may have had prior to entering this study. If you already have specimens that have been collected and stored, these may be requested in place of or in addition to new samples.
- CT scan: You will have a CT scan to ensure the disease is limited to the rectum.
- Blood tests: blood will be taken to confirm that blood counts and organ functions are within desired ranges and for research purposes.
  - The biopsy specimens and the blood tests will also be used to identify markers which may help predict the response to chemoradiation and to further study the cancer.
- Stool test: You will be asked to provide a small stool specimen (a sample of your bowel motion/poo) using the provided kit in the privacy of your own home. These specimens can be brought to your appointment or mailed to the hospital (postage will be provided).
- Questionnaires: In addition, you will be asked to complete a set of questionnaires which are explained on the next page.

Chemoradiation: After completing the screening process, you will have chemoradiation. This is the current standard treatment for your disease and you will receive the standard doses of radiation and chemotherapy.

Doublet chemotherapy: After completing the combined chemotherapy and radiotherapy course, you will then receive a combination of 2 chemotherapy drugs given in regular cycles over a 4-month period. This represents standard management (not research). This part of the treatment may not proceed, or may be stopped early, if you decide to stop or if your doctor recommends stopping.

Allocation: 2-3 weeks after finishing all the chemotherapy, you will be assessed by the study doctors. If you do not receive the doublet chemotherapy, or if you stop this early, then you will be assessed at a minimum of 8 weeks after completing radiotherapy. This assessment period is called the “*allocation”* process. You will have an examination and colonoscopy and biopsies from the cancer area and blood tests will be taken, you may also have an MRI.

Stool Test: You will be asked to provide a small stool specimen using the provided kit in the privacy of your own home. These specimens can be brought to your appointment or mailed to the hospital (postage will be provided).

Questionnaires: In addition, you will be asked to complete a set of questionnaires which are explained in the next page.

- If there are no signs of the cancer found, you will have an MRI scan. If the MRI scan does not show signs of cancer in the rectum, you will be allocated to the “Watch and Wait arm”.
- If there is any sign that there may still be some cancer present, you will be allocated to the *standard management* arm, and current best practice.

Watch and Wait Arm:

- After being allocated in this arm, your treating oncologist may recommend a course of chemotherapy. This usually takes around 3 months.
- Participants in the watch and wait arm will be monitored closely to detect any signs of cancer reappearing.
- During the monitoring period, if there are any signs of the cancer reappearing, the participant will be assessed by their surgeon with the goal of resecting the cancer, this will involve removing the rectum or part of the rectum.
- The monitoring process during the first year (from the allocation visit) includes:
  - Visits at each 3-month interval
    - Each visit will involve: asking you questions about your health, physical examination, sigmoidoscopy and biopsy and blood tests
  - MRI scans at each 3-month interval
  - CT scans at each 6-month interval
  - You will be asked to complete the study questionnaires at the end of the first year of monitoring
- The monitoring process during the second year (from the allocation visit) includes:
  - Visits at each 3-month interval
    - Each visit will involve: asking you questions about your health, physical examination, colonoscopy and biopsy
  - Blood tests and MRI scans at each 6-month interval
  - A CT scan at the completion of the second year
  - You will be asked to complete the study questionnaires at the end of the second year of monitoring

The 3-monthly MRI scans in the watch and wait arm will be covered by the grant funding on this study. You will not be asked to pay for these.

Standard Management Arm:

During the allocation process, if the assessments show that there are signs of cancer, you will be assessed by your surgeon with the goal of resecting the cancer; this will involve removing the rectum or part of the rectum. This is the current standard management for the disease. In the Standard management arm, you will be monitored according to the standard plan for patients with rectal cancer. In addition, you will be asked to complete the study questionnaires at the end of the first and the second year after the allocation visit.

Any assessments or scans that you have if allocated to the standard management arm would be performed even if you were not on this trial; any out-of-pocket expenses for these assessments will not be reimbursed or covered by the grant funding on this study.

**Will I have surgery?**

After completing the chemoradiation (CRT), participants will be assessed by the treating surgeon and treating doctors:

- Participants who have signs of cancer remaining in the bowel (Not complete clinical response [Not cCR])will proceed with the standard arm and will have surgery.

- Initially, participants who have a complete clinical response (cCR) will go into the Watch and Wait arm and will not have surgery and will be monitored closely: if any signs of cancer in the rectum are found during the monitoring phase, then they will proceed with surgery.

**What does the surgery involve?**

Surgery will involve removing the cancer and surrounding tissue to make sure no part of the cancer is left behind. Depending on the location of the cancer, the surgeon may be able to join the rectum together again to restore normal function. For some rectal cancers, the rectum cannot be rejoined and an artificial opening (colostomy) for body waste (faeces) is made in the wall of the abdomen. Depending on the location of the cancer and the type of surgery, the colostomy may be temporary (can be reversed) or may be permanent.

Questionnaires: Participation in this study involves answering 7 questionnaires at different times during the study. The questionnaires are provided in paper form. They should take about 30 minutes to complete.

You will be asked to answer all of the questions yourself by choosing the number that best applies to you. There are no "right" or "wrong" answers. The information that you provide will remain strictly confidential.

1. Questionnaires: (completing the questionnaires will take around 30 minutes). **Self-Administered Comorbidity Questionnaire (SCQ)** includes 14 questions; asks about common medical conditions. This will help us interpret the results of the study. You will be asked to complete this questionnaire in your first visit. This questionnaire takes about 5 minutes to complete.

You will be asked to complete the following questionnaires at your first visit, after completing the chemoradiotherapy and at one and two years after completing the chemoradiation. These questionnaires will take about 20 minutes to complete.

1. **Quality of Life Questionnaire (EORTC QLQ-C30)** includes 30 questions; assess the quality of life of participants with a history of cancer.
2. **Quality of Life in Colorectal Patients (EORTC QLQ-CR29)** includes 29 questions; assess the quality of life in participants with a history of colorectal cancer
3. **EQ-5D-5L Health-related quality of life (HRQoL)** includes 5 questions; assess different aspects of physical, mental, emotional and social functioning.
4. **MSKCC Bowel Function Inventory (MSKCC BFI)** and **Low Anterior Resection Syndrome (LARS)** **Score**: Incontinence and symptoms related to bowel function are some of the common problems that rectal cancer survivors experience. We will assess these issues using these two questionnaires. They include a total of 23 questions. [you will be asked to complete these questionnaires only if you do not have a colostomy]
5. **Fear of Recurrence Inventory (FCRI)** includes 9 questions about “Fear of the cancer recurrence”. Fear of the cancer recurrence (feeling anxious and frightened about the cancer coming back) is one of the issues reported by cancer survivors. This is an area of unmet needs for many cancer survivors. **Response efficacy** questions: includes 3 questions; and focuses on your opinion about your treatment.

You will be asked to complete the following questionnaire at one and two years after completing the chemoradiation.

1. Survivors of cancer sometimes report ongoing issues and the need for various services after the completion of treatment. **Cancer Survivors’ Unmet Needs (CaSUN)** includes 42 questions; assess a range of different issues which survivors of cancer may face. This questionnaire will take about 10 minutes to complete.

**There are no additional costs associated with participating in this research project.**

**You will not be paid.**

**4 What do I need to do?**

- Appointments: You will be asked to attend study appointments, during the appointments you will be assessed by the study doctors. Depending on the appointment, certain tests will be performed as explained above.
- Tests: including Blood tests, CT scans and MRIs needed for the study will be arranged by the study team for you.
- Stool test: Twice during this study, you will be asked to provide a small stool specimen: once during the screening visit and once after finishing chemoradiation during the allocation visit. You will be asked to provide the specimens using the provided kit from the privacy of your own home. Then the stool specimens can be brought to your appointment or mailed to the hospital (postage will be provided).
- Questionnaires: During some of your visits, you will be asked to answer the questions in the mentioned study questionnaires.

**5 Other relevant information about the research project**

This research project is a collaboration between the colorectal surgical unit and medical oncology unit at your hospital.

**6 Do I have to take part in this research project?**

Participation in any research project is voluntary. If you do not wish to take part, you do not have to. If you decide that you can take part and later change your mind, you are free to withdraw from the project at any stage.

If you do decide that you can take part, you will be given this Participant Information and Consent Form to sign and you will be given a copy to keep.

Your decision whether you can or cannot take part, or take part and then be withdrawn, will not affect your standard treatment, relationship with those treating you or relationship with your treating doctors. Depending on the stage of the disease, standard treatment for rectal cancer may include: Surgery, Radiotherapy and chemotherapy and then follow-up by your treating doctors. The follow up involves clinic visits which include physical examination, and may also include blood tests, CT scans and colonoscopies.

**7 What are the alternatives to participation?**

You do not have to take part in this research project to receive treatment at this hospital. You will still receive standard follow-up of your cancer by your usual doctor. Your study doctor will discuss these options with you before you decide whether to take part in this research project. You can also discuss the options with your local doctor.

**8 What are the possible benefits of taking part?**

This study may help better identify a group of patients with rectal cancer who do not need to have the unnecessary resection of the rectum. The knowledge gained from this study may also help design tests to improve the selection of treatment options for future patients.

**9 What are the possible risks and disadvantages of taking part?**

**Watch and Wait arm:** participants in this arm will be monitored closely for signs of cancer reappearing with the aim of resecting the rectum if the cancer reappears. Although previous studies have shown the safety of this method, it is possible that the cancer could reappear in a way that it cannot be resected anymore. This can happen in the rectum and/or in other parts of the body. If the cancer reappears and cannot be safely resected, then the disease cannot be cured with current available treatment. Although previous studies suggest this method is safe, the main aim of this study is to confirm the safety of the watch and wait method and to ensure that this risk is not higher than the current standard management.

**MRI scans:** There is no risk of exposure to radiation during an MRI procedure. However, due to the use of the strong magnet, MRI cannot be performed on patients with: implanted pacemakers, intracranial aneurysm clips, cochlear implants, certain prosthetic devices, implanted drug infusion pumps, neurostimulators, bone-growth stimulators, certain intrauterine contraceptive devices; or any other type of iron-based metal implants.

MRI cannot be used in the presence of internal metallic objects such as bullets or shrapnel, as well as surgical clips, pins, plates, screws, metal sutures, or wire mesh. If you are pregnant or suspect that you may be pregnant, you should notify your physician. Due to the potential for a harmful increase in the temperature of the amniotic fluid, MRI is not advised for pregnant patients. MRI generally is not advised for patients with epilepsy.

**Colonoscopy and sigmoidoscopy**: are usually safe procedures, and complications are rare but can occur:

- Bleeding can occur from biopsies or the removal of polyps, but it is usually minimal and can be controlled. The risk is reported to be less than 3 in 1000
- The colonoscope or sigmoidosope can cause a tear or hole in the colon. This is a serious problem, but it rarely happens. The risk is reported to be around 1 in 2000.
- It is possible to have side effects from the sedative medicines.

The other study tests and scans are not thought to add additional risks or disadvantages compared to the standard follow up tests and scans which are part of the standard management of the disease.

During the study, if you become upset or distressed as a result of participation in the research, the study doctor will be able to arrange for counselling or other appropriate support. Any counselling or support will be provided by qualified staff who are not members of the research project team. This counselling will be provided free of charge.

**10 What will happen to my test samples?**

As part of this study we will be collecting blood samples and tumour biopsies during the study. We ask your permission to analyse these samples extensively. We will be looking at different chemicals related to the cancer, including tumour DNA and RNA. These are the chemicals found inside the cancer cells. The aim is finding a way to predict the effects of treatment on the cancer. We are not testing for genetic (inherited) diseases. We also plan to use the cancer cells found in the biopsy specimens for special tissue cultures, which may help predict if the cancer is susceptible to certain treatments.

Whilst this study does involve some analysis of genetic material, it will not result in or enable identification of participants.

In the stool sample, we will be looking at the bacterial composition of the samples by testing for bacterial genetic material. Samples of your stool obtained for the purpose of this research project will be transferred to Flinders University and/or SAMHRI (The South Australian Health and Medical Research Institute), for further analysis. These samples will not contain information that can directly identify you, they will be de-identified.

We are also requesting permission to do further testing of your stored specimen in the future. By signing the consent form you agree to allow the researchers in this study to use your sample for research purposes related to this study in the future. Your identity will not be revealed and all samples will be coded. The link between the code and your personal details will be kept in password locked files.

Your samples will not be sold.

**11 What if new information arises during this research project?**

Sometimes during the course of a research project, new information becomes available about the treatment that is being studied. If this happens, the study doctor will tell you about it.

**12 Can I have other treatments during this research project?**

Your treating team will ask you about the prescribed medications, supplements and any herbal medications that you may be taking. You should continue all your prescribed treatments as usual unless otherwise advised. If you are taking supplemental folic acid, you will be asked to withhold it during the chemoradiation and during chemotherapy, as it may interact with some of the chemotherapy medications.

**13 Can I withdraw from this research project?**

If you decide to withdraw from this research project, please notify a member of the research team before withdrawal. If you do withdraw consent during the research project, the study doctor and relevant study staff will not collect additional personal information from you, although personal information already collected will be retained to ensure that the results of the research project can be measured properly and to comply with law. You should be aware that data collected up to the time you withdraw will form part of the research project results. If you do not want them to do this, you must tell them before you join the research project. If you withdraw from the study, you will continue to receive the best possible care and follow-up by your treating doctors.

**14 Could this research project be stopped unexpectedly?**

The results of the study will be reviewed regularly during the study period. If the study treatment approach (Watch and Wait) fails in a certain proportion of participants, then based on specified safety measures the study may close to recruitment earlier than expected. If this happens the study team will inform you of such event.

**15 What happens when the research project ends?**

Participants will continue with their regular follow-up arrangements after the project ends. Data collection for this project will only be during the study period.

When the research project is completed, participants will be provided with a summary of the results by their treating surgeon or medical oncologist.

**Part 2 How is the research project being conducted?**

**16 What will happen to my information?**

The data collected or used is coded and individually re-identifiable. The trial data will be kept in Flinders Medical Centre and in a protected area where only research staff will have access to. The data will be kept for a minimum 15 years post end of study.

By signing the consent form you consent to the study doctor and relevant research staff collecting and using personal information about you for the research project. Any information obtained in connection with this research project that can identify you will remain confidential. Your information will only be used for the purpose of this research project and it will only be disclosed with your permission, except as required by law.

Information about the participants may be obtained from their health records held at this and other health services, for the purpose of this research. By signing the consent form you agree to the research team accessing health records if they are relevant to participation in this research project.

It is anticipated that the results of this research project will be published and/or presented in a variety of forums. In any publication and/or presentation, information will be provided in such a way that the participants cannot be identified.

In accordance with relevant Australian and/or *South Australian* privacy and other relevant laws, you have the right to request access to the information collected and stored by the research team about you. You also have the right to request that any information with which you disagree be corrected. Please contact the research team member named at the end of this document if you would like to access your information.

Any information obtained for the purpose of this research that can identify you will be treated as confidential and securely stored. It will be disclosed only with your permission, or as required by law.

**17 Complaints and compensation**

If you suffer any injuries or complications as a result of this research project, you should contact the study team as soon as possible and you will be assisted with arranging appropriate medical treatment: If you are eligible for Medicare, you can receive any medical treatment required to treat the injury or complication, free of charge, as a public patient in any Australian public hospital.

**18 Who is organising and funding the research?**

This research project is being coordinated by: Flinders Medical Centre.

This study is sponsored by Southern Adelaide Local Health Network (SALHN) and partly funded by the Australasian Gastro-Intestinal Trials Group (AGITG) Innovation Grant.

This study will be coordinated by the Flinders Medical Centre Medical Oncology Clinical Trials Unit.

This study has an Independent Safety and Data Monitoring Committee. The Safety and Data Monitoring Committee will periodically review and evaluate the accumulated study data for participant safety, study conduct and progress, and, when appropriate, efficacy, and will make recommendations to the study team, concerning the continuation, modification, or termination of the trial. These reviews will be triggered when certain events occur. It is planned to have Safety and Data Monitoring Committee meetings at regular intervals during the study.

No member of the research team will receive a personal financial benefit from involvement in this research project (other than their ordinary wages).

**19 Who has reviewed the research project?**

All research in Australia involving humans is reviewed by an independent group of people called a Human Research Ethics Committee (HREC). The ethical aspects of this research project have been approved by the Southern Adelaide Clinical Human Research Ethics Committee. This project will be carried out according to the *National Statement on Ethical Conduct in Human Research (2007)*. This statement has been developed to protect the interests of people who agree to participate in human research studies.

**20 Further information and who to contact**

The person you may need to contact will depend on the nature of your query.

If you want any further information concerning this project or if you have any medical problems which may be related to involvement in the project (for example, any side effects), you can contact the principal study doctor on [contact number] or any of the following people:

**Clinical contact person**

| Name |  |
| --- | --- |
| Position |  |
| Telephone |  |
| Email |  |

For matters relating to research at the site at which the participant is taking part, the details of the local site complaints person are:

**Complaints contact person**

| Institution |  |
| --- | --- |
| Position |  |
| Telephone |  |
| Email |  |

If you have any complaints about any aspect of the project, the way it is being conducted or any questions about being a research participant in general, then you may contact:

**Reviewing HREC approving this research** **and HREC Executive Officer details**

| Reviewing HREC |  |
| --- | --- |
| Position |  |
| Telephone |  |
| Email |  |

**Local HREC Office contact (Single Site - Research Governance Officer)**

| Institution |  |
| --- | --- |
| Position |  |
| Telephone |  |
| Email |  |

**
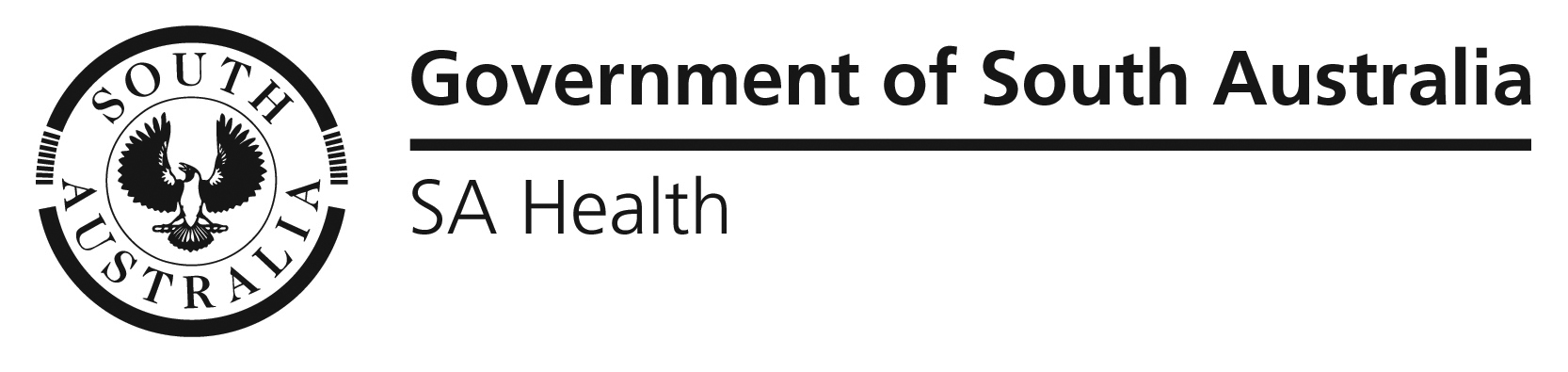
**

**Consent Form**

| **Title** | A Longitudinal Cohort Study of ‘Watch and Wait’ in Complete Clinical Responders after Chemoradiotherapy for Localised Rectal Cancer |
| --- | --- |
| **Short Title** | REctal cancer Non-Operative (RENO) |
| **Protocol Number** | 2020-3 |
| **Project Sponsor** | Southern Adelaide Local Health Network (SALHN) |
| **Principal Investigators** |  |
| **Location** | [study site] |

**Declaration by Participant**

- I have read the Participant Information Sheet or someone has read it to me in a language that I understand.
- I understand the purposes, procedures and risks of the research described in the project.
- I have had an opportunity to ask questions and I am satisfied with the answers I have received.
- I freely agree to taking part in this research project as described and understand that I am free to withdraw at any time during the project without affecting my future health care.
- I understand that I will be given a signed copy of this document to keep.
- I give permission for any previously collected tissue, blood or faecal samples to be used
- I give permission for my doctors, other health professionals, hospitals or laboratories outside this hospital to release information to [the study team] concerning my condition and treatment for the purposes of this project. I understand that such information will remain confidential.

|  |  |  | | | | | |  |
| --- | --- | --- | --- | --- | --- | --- | --- | --- |
|  | Name of Participant (please print) | | |  | | | |  |
|  |  | |  | | | | |  |
|  |  | | | |  | | |  |
|  | Signature | |  | | | Date |  |  |
|  | | | | | | | | |

*Under certain circumstances (see* Note for Guidance on Good Clinical Practice CPMP/ICH/135/95 at 4.8.9*) a witness* to informed consent is required*

|  | | | | | | | |
| --- | --- | --- | --- | --- | --- | --- | --- |
|  | Name of Witness* to Participant’s Signature (please print) | |  | | | |  |
|  |  |  | |  | | |  |
|  | Signature |  | | | Date |  |  |
|  | | | | | | | |

* Witness is not to be the investigator, a member of the study team or their delegate. In the event that an interpreter is used, the interpreter may not act as a witness to the consent process. Witness must be 18 years or older.

**Declaration by Study Doctor/Senior Researcher^†^**

I have given a verbal explanation of the research project, its procedures and risks and I believe that the person responsible for the participant has understood that explanation.

|  | | | | | | |
| --- | --- | --- | --- | --- | --- | --- |
|  | Name of Study Doctor/  Senior Researcher^†^  (please print) | |  | | |  |
|  | | | | | |  |
|  | Signature |  | | Date |  |  |
|  | | | | | | |

^†^ A senior member of the research team must provide the explanation of, and information concerning, the

research project.

Note: All parties signing the consent section must date their own signature.

**
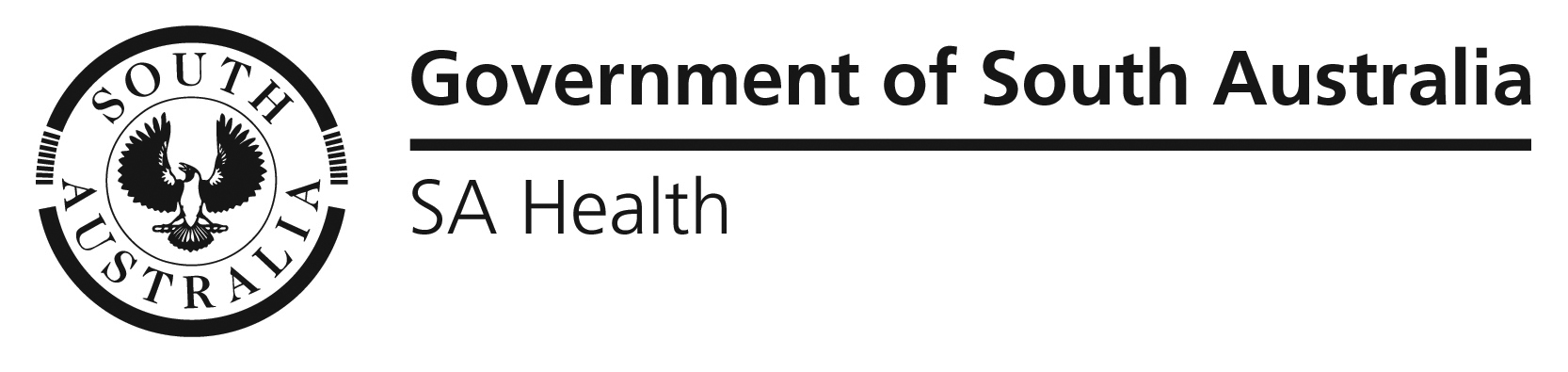
**

**Form for Withdrawal of Participation**

| **Title** | A Longitudinal Cohort Study of ‘Watch and Wait’ in Complete Clinical Responders after Chemoradiotherapy for Localised Rectal Cancer |
| --- | --- |
| **Short Title** | REctal cancer Non-Operative (RENO) |
| **Protocol Number** | 2020-3 |
| **Project Sponsor** | Southern Adelaide Local Health Network (SALHN) |
| **Principal Investigator** |  |
| **Location** | [study site] |

**Declaration by Participant**

I wish to withdraw from taking part in the above research project and understand that such withdrawal will not affect my routine treatment, relationship with those treating me or relationship with *[study site]*.

|  |  |  | | | | | |  |
| --- | --- | --- | --- | --- | --- | --- | --- | --- |
|  | Name of Participant (please print) | | |  | | | |  |
|  |  | |  | | | | |  |
|  |  | | | |  | | |  |
|  | Signature | |  | | | Date |  |  |
|  | | | | | | | | |

*In the event that the participant’s decision to withdraw is communicated verbally, the Study Doctor/Senior Researcher will need to provide a description of the circumstances below.*

|  |
| --- |

**Declaration by Study Doctor/Senior Researcher^†^**

I have given a verbal explanation of the implications of withdrawal from the research project and I believe that participant has understood that explanation.

|  | | | | | | |
| --- | --- | --- | --- | --- | --- | --- |
|  | Name of Study Doctor/  Senior Researcher^†^  (please print) | |  | | |  |
|  | | | | | |  |
|  | Signature |  | | Date |  |  |
|  | | | | | | |

^†^ A senior member of the research team must provide the explanation of, and information concerning, withdrawal from the research project.

Note: All parties signing the consent section must date their own signature.
