# Supplementary material for: A longitudinal cohort study of watch and wait in complete clinical responders after chemo-radiotherapy for localised rectal cancer: study protocol
Source: BMC Cancer. 2022 Mar 1;22:222. doi: 10.1186/s12885-022-09304-x (PMC8887187; doi:10.1186/s12885-022-09304-x)
Supplement: Supplementary file 2 — Additional file 2. [file 12885_2022_9304_MOESM2_ESM.docx]

Supplement file 2:

MRI scan protocol:

The report should include information on T3 depth of penetration, nodal status (N1/2/1c), EMVI status, Circumferential resection margin (CRM) status, mrTRG and DWI status.

TECHNIQUE

Coverage: L5/S1 to anal verge

The tumour and all mesorectal lymph nodes at and above the level of the tumour should be covered by high-resolution (HR) sequences.

Low tumours within 5 cm of the anal verge need imaging angled to the anal canal to assess the relationship of tumour to the levator ani muscles and anal sphincter complex.

Technique: Anterior saturation bands should be used.

Phase L-R can be useful in the axial images to reduce breathing artefact.

Preparation: An antiperistaltic agent (e.g hyoscine butylbromide) can be given to reduce artefact from adjacent bowel motion.

Patients may fast, but there is no other bowel preparation required.

Per-rectal fluid or gel should NOT be used

|  | Sequence | Notes |
| --- | --- | --- |
| All tumours | Axial large FOV T2 | To cover whole pelvis |
|  | Sagittal T2 | HR sequence (as defined below) |
|  | Axial oblique T2 HR | Angled to the centre of the tumour  Acquired voxel < 1.3 mm^3^  16–18 cm FOV, 3 mm slice thickness  0.6 mm x 0.6 mm in plane resolution* |
|  | Coronal oblique T2 HR |  |
|  | Optional HR T2 oblique | Parallel to sacrum to cover mesorectum up to 5 cm above upper border of tumour if needed |
|  | DWI  b values 50,500,1000 OR  b values 50, 400, 800 | Axial oblique through the tumour or previous site of tumour  3-4 mm slices |
| Low tumours‡ | Coronal oblique T2 HR | Angled to the anal canal  HR parameters as above |
|  | Axial oblique T2 HR |  |

FOV: field of view

‡ Reaching to anorectal junction

*Calculated using acquired matrix measurements.  Interpolated or zipped measurements do not give the required spatial resolution.
