# Supplementary material for: A longitudinal cohort study of watch and wait in complete clinical responders after chemo-radiotherapy for localised rectal cancer: study protocol
Source: BMC Cancer. 2022 Mar 1;22:222. doi: 10.1186/s12885-022-09304-x (PMC8887187; doi:10.1186/s12885-022-09304-x)
Supplement: Supplementary file 3 — Additional file 3. [file 12885_2022_9304_MOESM3_ESM.docx]

**Response Efficacy scale**

- The following questions, focus on your opinion about your treatment
- Please answer questions 1 to 3 by choosing only one box for each question.
  - By recurrence, we mean the possibility that the cancer could return or progress in the same place or in another part of the body.

(Adapted from Anderson, RB (2000) Vicarious and persuasive influences on efficacy expectations to perform breast self-examination. *Public Relations review* 26(1): 97-114)

|  | Strongly agree | Agree | Neither Agree or Disagree | Disagree | Strongly disagree |
| --- | --- | --- | --- | --- | --- |
| 1. I believe the watch and wait strategy is a worthwhile approach for me | ☐ | ☐ | ☐ | ☐ | ☐ |
| 1. I believe the watch and wait strategy is an effective approach for detecting the recurrence of rectal cancer | ☐ | ☐ | ☐ | ☐ | ☐ |
| 1. I believe the watch and wait strategy is an effective approach for reducing the risk of recurrent rectal cancer | ☐ | ☐ | ☐ | ☐ | ☐ |
